# Levosimendana e Fibrilação Atrial: Uma Metanálise de Ensaios Clínicos Randomizados

**DOI:** 10.36660/abc.20230856

**Published:** 2024-08-02

**Authors:** Huan Wan, Jihua Feng, Pan Ji, Wei Chen, Jianfeng Zhang

**Affiliations:** 1 Department of Emergency Medicine Guangxi Medical University Nanning Guangxi China Department of Emergency Medicine,The Second Affiliated Hospital of Guangxi Medical University, Nanning - Guangxi China

**Keywords:** Simendana, Fibrilação Atrial, Metanálise em Rede

## Abstract

**Fundamento:**

A fibrilação atrial (FA) é uma complicação prevalente associada à levosimendana; no entanto, permanece incerto se existem disparidades nos efeitos da levosimendana na FA não pós-operatória e pós-operatória.

**Objetivos:**

Este estudo teve como objetivo avaliar o efeito da levosimendana na FA não pós-operatória e pós-operatória conduzindo uma metanálise de ensaios clínicos randomizados (ECR).

**Métodos:**

PubMed, Embase, Biblioteca Cochrane e outras bases de dados foram pesquisadas. Pares de revisores identificaram ECRs que compararam levosimendana e placebo ou outras terapias, e os resultados relataram dados de eventos de FA. Foram utilizados modelos de efeitos aleatórios (com nível de significância de 5%).

**Resultados:**

Foram incluídos 29 ensaios elegíveis compreendendo 6.550 participantes, onze dos quais avaliaram a incidência de FA não pós-operatória e 18 incluíram FA pós-operatória. A análise revelou que a levosimendana elevou significativamente o risco de FA no grupo não pós-operatório (OR, 1,62; IC 95%: 1,19-2,20; p=0,002) e reduziu a incidência de FA no grupo pós-operatório (OR, 0,65; IC 95%: 0,44-0,96; p=0,03). A ocorrência de FA diminuiu mais significativamente em pacientes que usaram levosimendana após cirurgia cardíaca (OR, 0,53; IC 95%: 0,32-0,88; p=0,02) do que em pacientes que usaram levosimendana antes da cirurgia cardíaca (OR, 0,67; IC 95%: 0,42-1,06; p=0,09). O risco de FA foi significativamente elevado pela grande dose em bolus de levosimendana (dose em bolus ≥12 μg/kg) (OR, 1,44; IC 95%: 1,10-1,88; p=0,004) e diminuído pela pequena dose em bolus de levosimendana (dose em bolus <12 μg/kg) (OR, 0,64; IC 95%: 0,34-1,20; p=0,16).

**Conclusão:**

A levosimendana foi associada a um aumento da incidência de FA não pós-operatória. O emprego da levosimendana foi eficaz na prevenção da FA pós-operatória.

## Graphical abstract

**Figura Central:**
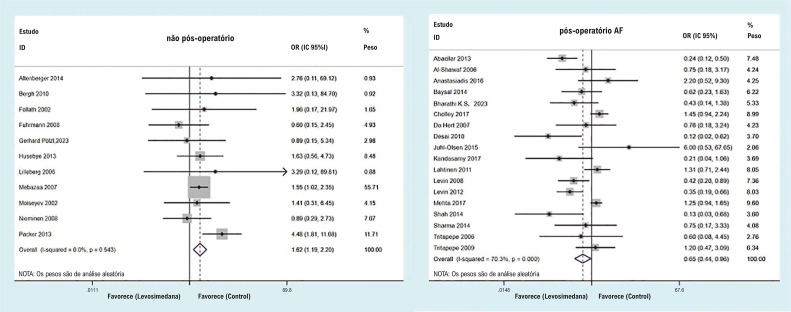
: Levosimendana e Fibrilação Atrial: Uma Metanálise de Ensaios Clínicos Randomizados

O risco de FA não pós-operatória e pós-operatória com Levosimendana.

## Introdução

A levosimendana é um sensibilizador de cálcio que melhora a contratilidade miocárdica e produz vasodilatação periférica. Eleva o débito cardíaco e tem impacto mínimo no consumo de oxigênio do miocárdio.^1^ Consequentemente, a levosimendana pode efetivamente melhorar as anormalidades hemodinâmicas em pacientes com IC. A levosimendana tem sido estudada em pacientes com insuficiência cardíaca (IC) congestiva e submetidos à cirurgia cardíaca.^2,3^ A levosimendana tem vários efeitos colaterais, incluindo pressão arterial baixa, dores de cabeça ou fibrilação atrial (FA), apesar do seu efeito positivo na IC.

A FA é prevalente em pacientes com IC. Vários fatores de risco são compartilhados entre FA e IC. Sua fisiopatologia é interdependente e, quando ocorrem simultaneamente, o risco de efeitos colaterais aumenta.^4,5^ A levosimendana reduz a demanda de oxigênio e melhora a contração miocárdica em pacientes com IC, o que pode indiretamente levar à redução da FA. Gaballah et al. descobriram que a levosimendana apresentou propriedades antiarrítmicas significativas em suas investigações de cardiomiócitos derivados de células pluripotentes induzidas em humanos.^6^ No entanto, alguns estudos demonstraram que a levosimendana também está associada a uma maior taxa de incidência de FA.^7,8^

O efeito da levosimendana nos dados de FA é inconsistente e, para avaliar isso, foi realizada uma metanálise compreendendo mais de 1.100 indivíduos de 14 ensaios clínicos randomizados (ECR). Nesses estudos, a levosimendana foi associada à diminuição da incidência de FA em comparação com os controles.^9^ Xing et al. conduziram em 2018 uma metanálise compreendendo 15 ECR para avaliar o efeito da levosimendana em pacientes com disfunção ventricular esquerda submetidos a cirurgia cardíaca e descobriram que não diminuiu a incidência de FA.^10^ Por outro lado, Jaguszewski et al. descobriram que a levosimendana aumenta significativamente o risco de FA.^11^ Os resultados contraditórios destes ensaios clínicos sublinham a necessidade de realizar mais investigação para avaliar o efeito da levosimendana no risco de FA. Mais importante ainda, o efeito da levosimendana na FA pós-operatória e não pós-operatória, a influência da fração de ejeção do ventrículo esquerdo (FEVE), a influência da dose da levosimendana, e a duração do acompanhamento dos dados de risco de FA não é clara. Assim, conduzimos esta metanálise, reunindo dados de todos os ECR disponíveis, que incluíram levosimendana e relataram FA como um evento adverso, para explorar ainda mais essas questões em detalhe.

## Métodos

### Critério de inclusão

Foram incluídos ECR que compararam levosimendana e placebo ou outras terapias e forneceram dados sobre a ocorrência de FA durante o acompanhamento.

### Métodos de pesquisa

PubMed, Embase, Biblioteca Cochrane até 1º de setembro de 2023, e outras bases de dados foram utilizadas para pesquisa. Além disso, os ensaios incluídos e as listas de referências de revisão relevantes foram pesquisados manualmente para encontrar mais artigos potencialmente elegíveis.

### Extração de dados e avaliação de qualidade

Em todos os ensaios incluídos, a FA não foi um objetivo pré-especificado. O número de eventos de FA foi estimado seguindo os dados de eventos adversos. Dois autores revisaram separadamente todos os títulos e resumos de artigos de pesquisa para eliminar estudos irrelevantes. As divergências sobre os dados extraídos foram resolvidas por discussão. Ferramentas da Cochrane Collaboration foram utilizadas para avaliar o risco de viés dos ECRs, que incluíram: ocultação de alocação, geração de sequência aleatória, dados de resultados incompletos, relato seletivo, cegamento e outros vieses.

### Pontos finais

Comparamos principalmente a incidência de FA não pós-operatória e pós-operatória entre os grupos levosimendana e controle. Os seguintes dados secundários foram avaliados: horário da infusão de levosimendana e dose de levosimendana.

### Análise estatística

Os dados dicotômicos foram calculados por intervalo de confiança (IC) de 95% e odds ratio (OR). Usamos um modelo de efeito aleatório. Gráficos de funil foram empregados para avaliar o viés de publicação. O software STATA 15.1 e o Review Manage versão 5.4 foram utilizados para as análises estatísticas. P<0,05 foi considerado estatisticamente significativo.

## Resultados

### Estudos elegíveis e características das disciplinas

**Figura 1:**
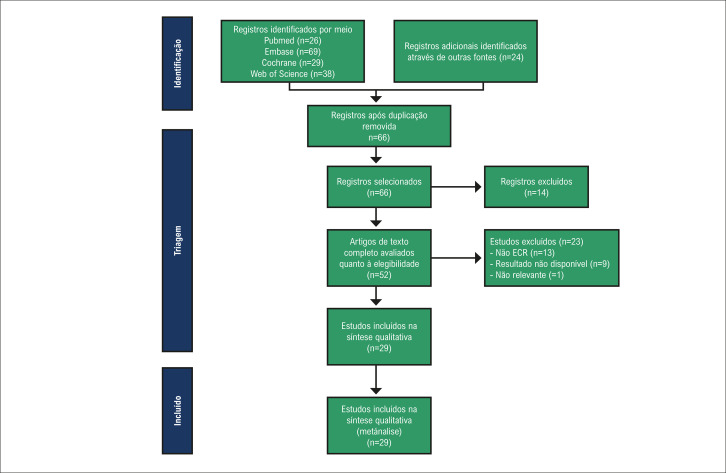
– Fluxograma da estratégia de seleção dos estudos. ECR: ensaios clínicos randomizados.

Foram identificados 186 estudos, dos quais 120 foram excluídos após triagem dos títulos e resumos. A Figura 1 apresenta o fluxograma de seleção. Vinte e nove estudos relataram dados sobre eventos de FA, abrangendo 6.550 participantes.^2,3,7,8,12-33^ Todos os ensaios foram desenhos de controle randomizado. As características detalhadas dos participantes estão resumidas na Tabela 1. Onze estudos realizaram estudos multicêntricos e os demais foram estudos unicêntricos. Em termos de agentes de controle, 19 estudos utilizaram placebo, cinco utilizaram dobutamina e cinco utilizaram terapia padrão.

**Tabela 1:** – Características dos estudos incluídos na metanálise

Estudo	Participantes(n)	População do estudo	FEVE média(%)		Dose em bolus	Dose de infusão contínua	Controle	Acompanhamento
			Levosimendana	Controle				
Abacilar 2013^19^	200	CRM	2 6,30 ± 6,36	24,86±1,08	Nenhum	24μg/kg	Placebo	No hospital
Al-Shawaf 2006^12^	30	CRM, FEVE≤35%	29 ± 6	31 ±6	12μg/kg	0,1-0,2 μg/kg/min por 24 horas	Milrinona	No hospital
Altenberger 2014^13^	120	AF, FEVE≤35%	24 ±5	24 ±5	Nenhum	0,2μg/kg/min por 6h em intervalos de 2 semanas durante 6 semanas	Placebo	24 semanas
Anastasiadis 2016^14^	32	CRM, FEVE≤40	35,7±4,9	37,5±3,4	Nenhum	0,1 μg/kg/min por 24 horas	Placebo	No hospital
Baysal 2014^15^	128	Cirurgia da valva mitral, FEVE≤45%	35,0 (20-50)	37,5 (25-50)	6μg/kg	0,1 μg/kg/min por 24 horas	Inotrópico padrão terapia	30 dias
Bergh 2010^2^	60	ICAD, FEVE≤35%	21,2±5,8	21,8±6,1	12μg/kg	0,1-0,2 μg/kg/min por 24 horas	Dobutamina	1 mês
Bharati 2023^47^	60	Cirurgia da valva mitral	-	-	Nenhum	0,1 mcg/kg/min após a indução por 24 horas	Placebo	6 horas, 24 horas e 7 dias
Cholley 2017^16^	335	CRM ou combinada com cirurgia valvar, FEVE≤40%	-	-	Nenhum	0,1 μg/kg/min por 24 horas	Placebo	6 meses
De Hert 2007^17^	30	Cirurgia cardíaca eletiva, FEVE≤30%	24±6	27±3	Nenhum	0,1μg/kg/min	Milrinona	No hospital
Desai 2018^18^	60	CRM sem CEC, FEVE <30%	25,17±5,49	25,5±4,42	Nenhum	0,1 μg/kg/min por 24 horas	Inotrópicos convencionais	No hospital
Follath 2002^20^	203	IC, FEVE <35%	-	-	24μg/kg	0,1 μg/kg/min por 24 horas	Dobutamina	No hospital
Fuhrmann 2008^21^	32	Choque cardiogênico	22(18-31)/22±9	27(20-34)/27±10	12μg/kg	0,1-0,2 μg/kg/min por 24 horas	Enoximona	30 dias
Pölzl 2023^33^	145	IC,FEVE≤30%	24±5	24±5	Nenhum	0,2 mcg/kg/min a cada 2 semanas ou como infusão de 24 horas a uma taxa de 0,1 mcg/kg/min a cada 3 semanas	Placebo	14 e 26 semanas
Husebye 2013^22^	61	IC dentro de 48 horas após um IAMCSST tratado com ICP primária	43 (38-49)	40 (33-47)	12μg/kg	0,1 μg/kg/min por 24 horas	Placebo	6 meses
Juhl-Olsen 2015^23^	20	SVA, FEVE> 45%	62 (55–75)	62 (58–70)	Nenhum	0,1μg/kg/min	Placebo	No hospital
Kandasamy 2017^24^	80	CRM sem CEC,	-	-	Nenhum	0,1 μg/kg/min por 24 horas	Dobutamina	No hospital
Lahtinen 2011^3^	200	Cirurgia valvular ou combinada com CRM	77%>50%	73%>50%	24μg/kg	0,1μg/kg/min	Placebo	No hospital
Levin 2008^48^	137	Cirurgia coronária com LCOS	36,62±4,36	38,22±5,24	10μg/kg	0,1 μg/kg/min por 24 horas	Dobutamina	No hospital
Levin 2012^49^	252	CRM, FEVE<25%	17,56±3,24	18,62±2,12	10μg/kg	0,1 μg/kg/min por 24 horas	Placebo	No hospital
Lilleberg 2006^25^	22	IC	25±5	28±6	12μg/kg	0,1-0,2 μg/kg/min por 24 horas	Placebo	14 dias
Mebazaa 2007^8^	1320	ICAD	24±5	24±5	12μg/kg	0,1 μg/kg/min por 24 horas	Dobutamina	6meses
Mehta 2017^26^	849	Cirurgia cardíaca, FEVE<35%	26(24–32)	27(22-31)	12μg/kg	0,1 μg/kg/min por 24 horas	Placebo	30 dias
Moiseyev 2002^27^	504	Insuficiência ventricular esquerda	-	-	6-24μg/kg	0,1-0,4μg/kg/min	Placebo	No hospital
Nieminen 2008^28^	307	IC, FEVE≤30%	25±5,3	25±4,9	Nenhum	Cápsula de 1 mg uma ou duas vezes ao dia	Placebo	180 dias
Packer 2013^7^	587	ICAD	23±7	24±7	12μg/kg	0,1μg/kg/min	Placebo	No hospital
Shah 2014^29^	50	CRM sem CEC, FEVE<30%	22,45±4,06	22,56±3,41	Nenhum	200 μg/kg em 24 horas	Placebo	No hospital
Sharma 2014^30^	40	CRM+reparo da válvula mitral	23,55±4,87	22,55±0,92	Nenhum	200 μg/kg em 24 horas	Placebo	No hospital
Tritapepe 2006^31^	24	CRM	50±7	52±5	24μg/kg	Nenhum	Placebo	No hospital
Tritapepe 2009^32^	102	CRM	41,6±10,7	44,1±9,8	24μg/kg	Nenhum	Placebo	No hospital

FEVE: fração de ejeção do ventrículo esquerdo; CRM: cirurgia de revascularização do miocárdio; IC: insuficiência cardíaca; ICAD: insuficiência cardíaca agudamente descompensada; CRM sem CEC: cirurgia de revascularização do miocárdio sem circulação extracorpórea; IAMCSST: infarto com elevação do segmento ST; SVA: troca valvar aórtica; LCOS: síndrome de baixo débito cardíaco. Todos os estudos consideraram valores < 0,05 para indicar significância estatística.

### Qualidade do estudo e viés de publicação

**Figura 2:**
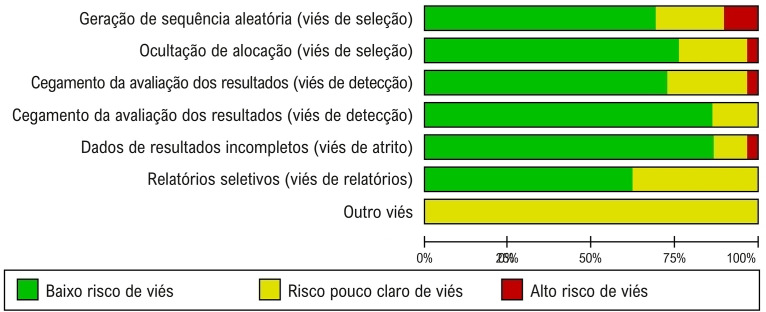
– Avaliação do risco de viés dos estudos incluídos.

**Figura 3:**
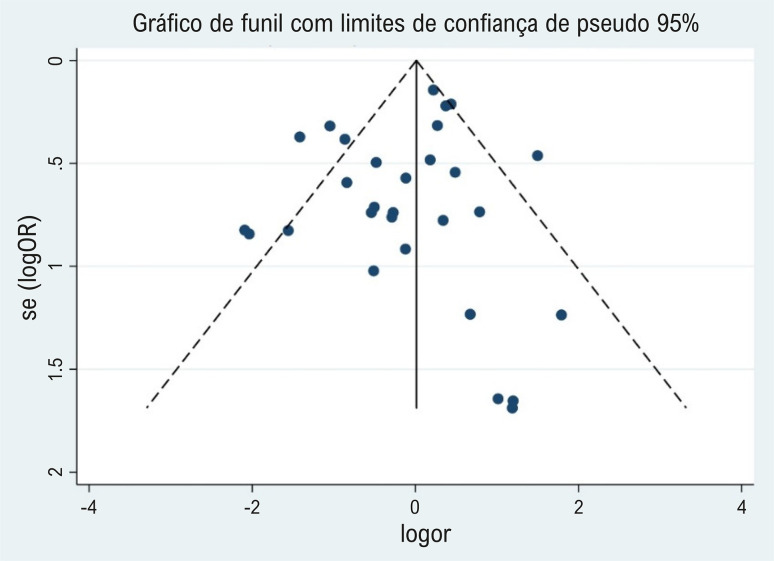
– Risco de viés de publicação dos estudos incluídos.

Vinte estudos foram randomizados usando métodos que incluíam cronogramas de randomização gerados por computador ou tabelas de números aleatórios, e o processo de randomização foi documentado. Em 22 estudos, a ocultação da alocação foi documentada com baixo risco de viés, mas isto foi questionável em outros sete. Em 73% dos estudos, foi utilizado duplo-cego, proporcionando um cegamento eficaz para limitar o preconceito dos participantes ou investigadores nos relatos de reações adversas. Os resultados da avaliação de risco de viés são apresentados na Figura 2. A Figura 3 ilustra o risco de viés de publicação nos estudos incluídos. O teste de Egger não revelou evidências de viés de publicação (p=0,251).

### Incidência de FA e levosimendana

Onze estudos avaliaram a ocorrência de FA não pós-operatória e afirmaram que o levosimendana elevou significativamente o risco de FA não pós-operatória (Figura Central). Dezoito estudos relataram que a levosimendana reduziu significativamente a incidência de FA pós-operatória (Figura Central). Além disso, a incidência de FA diminuiu significativamente mais em pacientes que usaram levosimendana após cirurgia cardíaca do que aqueles que usaram levosimendana antes da cirurgia cardíaca (Tabela 2). A heterogeneidade deveu-se principalmente a diferenças nos critérios de inclusão, dose de levosimendana, FEVE, cirurgia cardíaca e duração do acompanhamento.

**Tabela 2:** – Análises de subgrupos

Grupo	No. estudos	Heterogeneidade		Modelo	Metanálise	
		p	I^**2**^(%)		OR (IC95%)	p
**Levosimendana vs. Placebo/Controle**						
FEVE≤40%	20	<0,001	71,6	Aleatório	0,82 (0,55, 1,22)	0,33
FEVE>40%	4	0.545	0	Aleatório	1,30 (0,80, 2,13)	0,30
**Tempo de administração**						
Antes da cirurgia	14	<0,001	75,3	Aleatório	0,67 (0,42, 1,06)	0,09
Depois da cirurgia	4	0,870	0	Aleatório	0,53 (0,32, 0,88)	0,02
**Duração do acompanhamento**						
≤7 dias	18	<0,001	68,5	Aleatório	0,68 (0,42,1.12)	0,14
>7 dias	11	0.812	0	Aleatório	1,30 (1,07, 1,57)	0,007
**Dose em bolus**						
≥12 μg/kg	10	0.294	16.2	Aleatório	1,44 (1,10, 1,88)	0,004
<12 μg/kg	9	<0,001	72,6	Aleatório	0,64 (0,34, 1,20)	0,16

*FEVE: fração de ejeção do ventrículo esquerdo.*

### Análise de subgrupo

Foi realizada análise de subgrupos de acordo com a FEVE: FEVE≤40% e FEVE>40%. A levosimendana não alterou significativamente a incidência de FA em ambos os subgrupos (Tabela 2).

A levosimendana foi administrada em bolus seguida de infusão contínua em 14 estudos, enquanto apenas infusão contínua foi utilizada em 13 estudos nesta metanálise. A dose em bolus variou de 6 a 24 μg/kg, e a dose de infusão contínua variou de 0,1 a 0,4 μg/kg/min. O efeito da dose em bolus de levosimendana no risco de FA foi avaliado através da realização de outra análise de subgrupo. Os participantes foram classificados em dois subgrupos: uma grande dose em bolus (dose de levosimendana ≥12 μg/kg) e uma pequena dose em bolus (dose de levosimendana <12μg/kg) com 0,1 e 0,2 μg/kg/min em infusão contínua. A incidência de FA foi significativamente aumentada por uma grande dose em bolus e diminuída por uma pequena dose em bolus (Tabela 2).

Analisando a duração do acompanhamento, a levosimendana foi significativamente associada a um alto risco de FA em onze estudos com mais de sete dias de acompanhamento. Em 18 estudos com seguimento não superior a sete dias, foi observada menor incidência de FA no grupo levosimendana em comparação aos controles (Tabela 2).

### Análise da sensitividade

**Figura 4:**
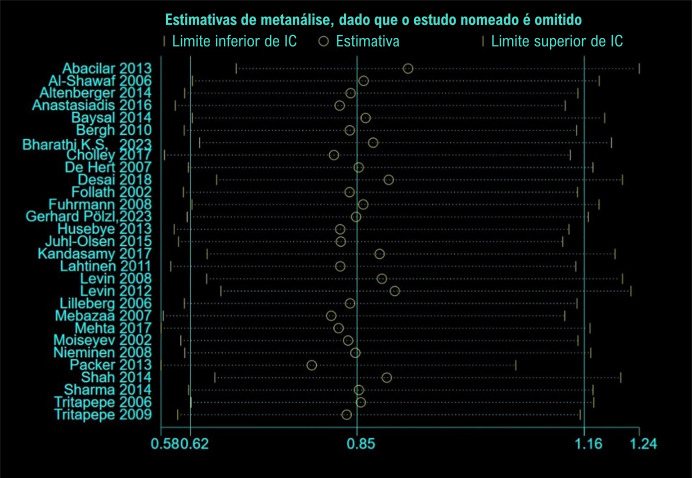
– Análise de sensibilidade.

Os resultados agrupados foram calculados. Nenhum dos estudos influenciou os resultados agrupados e alterou a conclusão desta análise. Os resultados agrupados do estudo foram análogos (Figura 4).

## Discussão

Até onde sabemos, esta metanálise continha o maior número de ECR, que compararam levosimendana e placebo ou outras terapias e forneceram dados sobre a ocorrência de FA durante o acompanhamento. De acordo com nossos achados, altas incidências de FA não pós-operatória e baixas incidências de FA pós-operatória foram associadas à levosimendana. Descobrimos ainda que os pacientes que usaram levosimendana após cirurgia cardíaca tiveram uma diminuição mais pronunciada na incidência de FA do que aqueles que usaram levosimendana antes da cirurgia cardíaca.

A levosimendana é um sensibilizador de cálcio que aumenta a contratilidade miocárdica, aumentando a sensibilidade do miofilamento ao cálcio através da ligação à troponina C cardíaca de maneira dependente do cálcio.^34^ Também abre canais de potássio mitocondriais sensíveis ao ATP nos tecidos cardíacos e vasculares e exerce efeitos vasodilatadores periféricos e anti-isquêmicos.^35^ Agentes inotrópicos comuns melhoram a contratilidade miocárdica aumentando o Ca2+ que pode se ligar à troponina-C cardíaca aumentando a energia miocárdica, a demanda de oxigênio e a incidência de arritmia. Portanto, tem potencial para causar arritmias. Por outro lado, a levosimendana não aumenta a demanda de energia miocárdica e o consumo de oxigênio.^36^ Não afeta o cálcio livre intracelular, portanto, o potencial para arritmias também é reduzido.

Uma metanálise anterior não achou que a infusão de levosimendana pudesse reduzir o risco de FA em pacientes submetidos a cirurgia cardíaca.^10^ Os ECR em larga escala (LEVO-CTS, CHEETAH e LICORN) também não aumentaram a taxa de incidência de FA no grupo levosimendana. Nossas descobertas demonstraram que a levosimendana diminuiu significativamente o risco de FA após cirurgia cardíaca e aumentou a incidência de FA não pós-operatória. A levosimendana diminuiu significativamente o aAPD90 e o aERP. A reentrada e a estabilidade da FA são afetadas pela redução da duração do potencial de ação e do período refratário.^37^ As características da cirurgia cardíaca diferem na levosimendana em pacientes com IC aguda. Os fatores aplicáveis aos pacientes cardiopatas costumam ser diferentes daqueles aplicáveis ao período perioperatório. A suspensão do tratamento perioperatório padrão, a perda sanguínea, o uso de circulação extracorpórea, a rápida transferência de fluidos intravasculares e extravasculares e a síndrome da resposta inflamatória sistêmica podem influenciar o resultado da terapia com levosimendana. O metabólito OR-1896 da levosimendana se forma lentamente no ambiente de cirurgia cardíaca em comparação com pacientes com IC. Os níveis máximos foram alcançados cinco a seis dias após a interrupção da infusão de levosimendana devido ao jejum após a cirurgia e ao uso de antibióticos de amplo espectro em cirurgia cardíaca.^38^ Pelas características farmacocinéticas da levosimendana, seu uso antes ou durante o período perioperatório pode ter efeitos muito diferentes. As características inotrópicas e cardioprotetoras da levosimendana podem ter efeito duradouro, auxiliando na redução de complicações pós-operatórias. Especialistas aconselharam o uso de levosimendana para pacientes com função miocárdica geralmente prejudicada um dia antes da cirurgia cardíaca.^39^ Contudo, a análise de subgrupo mostrou que o uso de levosimendana após cirurgia cardíaca é ligado a uma diminuição considerável na incidência de FA. O mecanismo de inibição da FA pós-operatória pela levosimendana não é claro, mas pode estar relacionado às características antioxidantes e anti-inflamatórias da levosimendana.^40^ No entanto, atualmente não existem ensaios de qualidade significativamente alta utilizando levosimendana nesse momento, exigindo uma avaliação adicional.

A taxa de incidência de FA aumenta com a gravidade da IC.^41^ Harrison et al. conduziram uma metanálise compreendendo 14 ECR, incluindo 1.155 pacientes de cirurgia cardíaca agrupados por FEVE,^9^ para explorar o perfil de segurança e eficácia da levosimendana. Os pacientes foram classificados em grupos com FEVE baixa ou preservada, com média de FEVE< 40% ou FEVE> 40%, respectivamente. A análise dos dados demonstrou uma diminuição significativa no risco de morte com levosimendana, e a análise de subgrupo mostrou que isto se limitou apenas aos estudos de baixa FEVE. Nenhum benefício foi observado no “grupo com FEVE preservada”. Análises adicionais revelaram que os pacientes do “grupo de baixa FEVE” que receberam levosimendana diminuíram significativamente a incidência de FA pós-operatória. O grupo com FEVE preservada também não apresentou diferença. Nossos achados demonstraram que a levosimendana não alterou significativamente a incidência de FA nos subgrupos FEVE>40% e FEVE≤40%.

Um estudo anterior mostrou que uma dose em bolus de 6-24 μg/kg administrada em 10 minutos seguida por uma taxa de infusão de 0,05 a 0,2 μg/kg/min é o regime de dosagem ideal de levosimendana.^42^ Papp et al. sugeriram outro regime de levosimendana, 6-12μg/kg entregue em 10 min seguido por 0,05 ou 0,1 ou 0,2μg/kg/min por 24h.^43^ Como a dobutamina tem meia-vida de poucos minutos, enquanto a meia-vida da levosimendana é de cerca de uma hora, os efeitos hemodinâmicos da dobutamina podem ser observados imediatamente após a infusão, enquanto o efeito imediato pode ser observado apenas com uma grande dose de levosimendana. Altas doses de levosimendana foram associadas a arritmias em casos de hipovolemia ou hipotensão inicial.^44,45^ Nossos achados demonstraram que uma grande dose em bolus de levosimendana (≥12 μg/kg) estava associada a uma alta incidência de FA, e uma pequena dose em bolus de levosimendana (<12 μg/kg) estava associada a uma baixa incidência de FA. Os especialistas do Consenso Europeu sobre o uso de levosimendana durante o período perioperatório não recomendam a administração de altas doses. Quando é necessária a administração de altas doses, a maioria recomenda reduzir a dose.^39^

Nieminen et al. demonstraram que a administração intravenosa de levosimendana por 24 horas leva a efeitos hemodinâmicos dose-dependentes, estabelecendo uma correlação clara entre eles. Seus metabólitos ativos ligados às proteínas OR-1855 e OR-1896 exercem efeitos clínicos por até sete dias.^46^ A presença do metabólito de ação prolongada significa que os efeitos hemodinâmicos continuam por uma semana após a infusão de levosimendana, e a possibilidade de reações adversas também aumenta com o tempo. Esta metanálise de pesquisa revelou que a levosimendana estava significativamente ligada a um alto risco de FA com mais de sete dias de acompanhamento. A levosimendana tendeu a aumentar o risco de FA quando o seguimento foi menor ou igual a sete dias. Um grande estudo randomizado e controlado é necessário.

Até onde sabemos, esta é a primeira pesquisa a analisar a correlação de risco de levosimendana e FA com base no momento da administração, na dose de levosimendana e na duração do acompanhamento. Analisamos rigorosamente a literatura e incluímos todos os ECRs elegíveis contendo dados de levosimendana e FA. Esta pesquisa teve várias limitações. Primeiro, algumas avaliações de resultados tiveram heterogeneidade moderada ou alta, por isso fizemos muitas análises de subgrupos para cada resultado para investigar a fonte da heterogeneidade. Adicionalmente, fizemos uma análise de sensibilidade através da exclusão de estudos de alto risco, o que não alterou os resultados principais. Em segundo lugar, porque o tamanho da amostra de alguns estudos é muito pequeno, o que não traz evidências suficientes, são necessários grandes ensaios clínicos com evidências convincentes para resolver esta situação.

## Conclusões

A alta FA não pós-operatória e a baixa FA pós-operatória foram associadas à levosimendana, de acordo com a metanálise realizada neste estudo. Nossos achados demonstraram que uma grande dose em bolus de levosimendana estava associada a uma alta incidência de FA, e uma pequena dose em bolus de levosimendana estava associada a uma baixa incidência de FA. O uso de levosimendana estava consideravelmente ligado a um alto risco de FA com mais de sete dias de acompanhamento. A levosimendana tendeu a aumentar o risco de FA quando o seguimento foi menor ou igual a sete dias. Mais estudos clínicos de alta qualidade são necessários para confirmar nossos achados.
