# Supplementary material for: Levosimendana e Fibrilação Atrial: Uma Metanálise de Ensaios Clínicos Randomizados
Source: Arq Bras Cardiol. 2024 Aug 2;121(7):e20230856. [Article in Portuguese] doi: 10.36660/abc.20230856 (PMC11464093; doi:10.36660/abc.20230856)
Supplement: Translation

# Translation: Levosimendan and Atrial Fibrillation: A Meta-Analysis of Randomized Controlled Trials

**DOI:** 10.36660/abc.20230856i

**Authors:** Huan Wan, Jihua Feng, Pan Ji, Wei Chen, Jianfeng Zhang

**Affiliations:** 1 China Department of Emergency Medicine,The Second Affiliated Hospital of Guangxi Medical University, Nanning – Guangxi China

**Keywords:** Simendan, Atrial Fibrillation, Network Meta-Analysis

## Abstract

**Background:**

Atrial fibrillation (AF) is a prevalent complication associated with levosimendan; however, it remains uncertain whether there are any disparities in the effects of levosimendan on non-postoperative and postoperative AF.

**Objectives:**

This study aimed to evaluate the levosimendan effect on non-postoperative and postoperative AF by conducting a meta-analysis of randomized control trials (RCTs).

**Methods:**

PubMed, Embase, Cochrane Library, and other databases were searched. Pairs of reviewers identified RCTs that compared levosimendan and placebo or other therapies, and the results reported AF events data. Random effects models were used (at a significance level of 5%).

**Results:**

Twenty-nine eligible trials comprising 6550 participants were included, eleven of which evaluated the non-postoperative AF incidence, and 18 included postoperative AF. The analysis revealed that levosimendan elevated the AF risk significantly in the non-postoperative group (OR, 1.62; 95% CI: 1.19-2.20; p=0.002) and reduced the AF incidence in the postoperative group (OR, 0.65; 95% CI: 0.44-0.96; p=0.03). AF occurrence decreased more significantly in patients who used levosimendan after cardiac surgery (OR, 0.53; 95% CI: 0.32-0.88; p=0.02) than in patients who used levosimendan before cardiac surgery (OR, 0.67; 95% CI: 0.42-1.06; p=0.09). Moreover, The AF risk was significantly elevated by levosimendan large bolus dose (bolus dose≥12 μg/kg) (OR, 1.44; 95% CI: 1.10-1.88; p=0.004) and decreased by small bolus dose of levosimendan (bolus dose<12 μg/kg) (OR, 0.64; 95% CI: 0.34-1.20; p=0.16).

**Conclusion:**

Levosimendan was linked to an increased non-postoperative AF incidence. The employment of levosimendan was effective in preventing postoperative AF.

## Introduction

Levosimendan is a calcium-sensitizer that improves myocardial contractility and produces peripheral vasodilatation. It elevates the cardiac output and has a minimum impact on myocardial oxygen consumption.^1^ Accordingly, levosimendan can effectively improve hemodynamic abnormalities in heart failure (HF) patients. Levosimendan has been studied in patients with congestive HF and those undergoing cardiac surgery.^2,3^ Levosimendan has various side effects, including low blood pressure, headaches, or atrial fibrillation (AF) despite its positive effect on HF.

AF is prevalent in cardiac failure patients. Various risk factors are shared between AF and HF. Their pathophysiology is interdependent, and when they concurrently occur, the side effects risk increases.^4,5^ Levosimendan reduces oxygen demand and improves myocardial contraction in HF patients, which may indirectly lead to a reduction in AF. Gaballah et al. found that levosimendan showed significant antiarrhythmic properties in their investigation of cardiomyocytes derived from induced pluripotent cells in humans.^6^ However, some studies have shown that levosimendan is also associated with a higher incidence rate of AF.^7,8^

The levosimendan effect on AF data is inconsistent, and to assess this, a meta-analysis comprising over 1100 individuals from 14 randomized controlled trials was conducted. In those studies levosimendan was linked to AF incidence decrease in comparison with controls.^9^ Xing Z et al. conducted in 2018 a meta-analysis comprising 15 randomized controlled trials to assess the levosimendan effect in left ventricular dysfunction patients having cardiac surgery and found that it did not decrease the AF incidence.^10^ Conversely, Jaguszewski et al. found that levosimendan significantly increases the risk of AF.^11^ These contradictory clinical trials outcome underlines the necessity to do more research to evaluate the levosimendan effect on AF risk. More importantly, the levosimendan effect in postoperative and non-postoperative AF, the left ventricular ejection fraction (LVEF) influence, the levosimendan dose, and the follow-up duration on the AF risk data are unclear. Thus, we conducted this meta-analysis, pooling data from all available randomized controlled trials (RCTs), which included levosimendan and reported AF as an adverse event, to further explore these questions in detail.

## Methods

### Inclusion Criteria

RCTs that compared levosimendan and placebo or other therapies and provided data on the AF occurrence during follow-up were included.

### Search methods

PubMed, Embase, Cochrane Library until 1^st^ September 2023, and other databases were used for research. Additionally, the included trials and relevant review reference lists were manually searched to find more potential eligible articles.

### Data extraction and quality assessment

In all the trials included, AF was not a pre-specified objective. The AF events number was estimated following the adverse event data. Two authors separately reviewed all research paper titles and abstracts to eliminate irrelevant studies. Disagreements on the extracted data were settled by discussion. Tools from the Cochrane Collaboration were utilized to evaluate the RCTs’ bias risk, which included: allocation concealment, random sequence generation, incomplete outcome data, selective reporting, blinding, and other biases.

### Endpoints

We primarily compared non-postoperative and postoperative AF incidence between levosimendan and control groups. The following secondary data were evaluated: time of levosimendan infusion and levosimendan dose.

### Statistical analysis

Dichotomous data were calculated by 95% confidence interval (CI) and odds ratios (ORs). We used a random-effect model. Funnel plots were employed to assess publication bias. STATA 15.1 software and Review Manage version 5.4 was used for the statistical analyses. P<0.05 was considered statistically significant.

## Results

### Eligible studies and subject characteristics

We identified 186 studies, 120 of which were excluded after screening the titles and abstracts. Figure 1 displays the selection flow chart. Twenty-nine studies reported data on AF events, covering 6550 participants.^2,3,7,8,12-34^ All trials were randomized control designs. The detailed participants’ characteristics are summarized in Table 1. Eleven trials performed multi-center studies, and the others were single-center studies. In terms of control agents, 19 studies used a placebo, five used dobutamine, and five used standard therapy.

**Figure 1:** – Literature search flow diagram.

**Table 1:** – Characteristics of included studies in the meta-analysis

Study	Participants(n)	Study population	Mean LVEF(%)		Bolus dose	Continous infusion dose	Control	Follow-up
			Levosimendan	Control				
Abacilar 2013^19^	200	CABG	2 6.30 ± 6.36	24.86 ± 1.08	None	24μg/kg	Placebo	In-hospital
Al-Shawaf 2006^12^	30	CABG, LVEF≤35%	29 ± 6	31 ±6	12μg/kg	0.1-0.2μg/kg/min for 24 hours	Milrinone	In-hospital
Altenberger 2014^13^	120	HF, LVEF≤35%	24 ±5	24 ±5	None	0.2μg/kg/min for 6h at 2-week intervals over 6 weeks	Placebo	24 weeks
Anastasiadis 2016^14^	32	CABG, LVEF≤40	35.7 ± 4.9	37.5 ± 3.4	None	0.1μg/kg/min for 24 hours	Placebo	In-hospital
Baysal 2014^15^	128	mitral valve surgery, LVEF≤45%	35.0 (20-50)	37.5 (25-50)	6μg/kg	0.1μg/kg/min for 24 hours	Standard inotropic therapy	30days
Bergh 2010^2^	60	ADHF, LVEF≤35%	21.2±5.8	21.8±6.1	12μg/kg	0.1-0.2μg/kg/min for 24 hours	Dobutamine	1 month
Bharathi, 2023^47^	60	mitral valve surgery	-	-	None	0.1 mcg/kg/min after induction for 24 hours	Placebo	6 hours, 24 hours, and 7 days
Cholley 2017^16^	335	CABG or combined with valve surgery, LVEF≤40%	-	-	None	0.1μg/kg/min for 24 hours	Placebo	6 months
De Hert 2007^17^	30	elective cardiac surgery, LVEF≤30%	24±6	27±3	None	0.1μg/kg/min	Milrinone	In-hospital
Desai 2018^18^	60	OPCAB, LVEF <30%	25.17±5.49	25.5±4.42	None	0.1μg/kg/min for 24 hours	Conventional inotropes	In-hospital
Follath 2002^20^	203	HF, LVEF <35%	-	-	24μg/kg	0.1μg/kg/min for 24 hours	Dobutamine	In-hospital
Fuhrmann 2008^21^	32	Cardiogenic shock	22(18-31)/22±9	27(20-34)/27±10	12μg/kg	0.1-0.2μg/kg/min for 24 hours	Enoximone	30 days
Gerhard Pölzl,2023^33^	145	HF, LVEF≤30%	24 ± 5	24 ± 5	None	0.2 mcg/kg/min every 2 weeks, or as 24-hour infusion at a rate of 0.1 mcg/kg/min every 3 weeks	Placebo	14 and 26 weeks
Husebye 2013^22^	61	HF within 48 h after a primary PCI-treated STEMI	43 (38-49)	40 (33-47)	12μg/kg	0.1μg/kg/min for 24 hours	Placebo	6 months
Juhl-Olsen 2015^23^	20	AVR, LVEF> 45%	62 (55–75)	62 (58–70)	None	0.1μg/kg/min	Placebo	In-hospital
Kandasamy 2017^24^	80	OPCABG	-	-	None	0.1μg/kg/min for 24 hours	Dobutamine	In-hospital
Lahtinen 2011^3^	200	Valve surgery or combined with CABG	77%>50%	73%>50%	24μg/kg	0.1μg/kg/min	Placebo	In-hospital
Levin 2008^48^	137	Coronary surgery with LCOS	36.62±4.36	38.22±5.24	10μg/kg	0.1μg/kg/min for 24 hours	Dobutamine	In-hospital
Levin 2012^49^	252	CABG, LVEF<25%	17.56±3.24	18.62±2.12	10μg/kg	0.1μg/kg/min for 24 hours	Placebo	In-hospital
Lilleberg 2006^25^	22	HF	25±5	28±6	12μg/kg	0.1-0.2μg/kg/min for 24 hours	Placebo	14 days
Mebazaa 2007^8^	1320	ADHF	24±5	24±5	12μg/kg	0.1μg/kg/min for 24 hours	Dobutamine	6 months
Mehta 2017^26^	849	Cardiac surgery, LVEF<35%	26(24–32)	27(22-31)	12μg/kg	0.1μg/kg/min for 24 hours	Placebo	30 days
Moiseyev 2002^27^	504	Left ventricular failure	-	-	6-24μg/kg	0.1-0.4μg/kg/min	Placebo	In-hospital
Nieminen 2008^28^	307	HF, LVEF≤30%	25±5.3	25±4.9	None	1 mg capsule once or twice daily	Placebo	180 days
Packer 2013^7^	587	ADHF	23±7	24±7	12μg/kg	0.1μg/kg/min	Placebo	In-hospital
Shah 2014 ^29^	50	OPCABG, LVEF<30%	22.45±4.06	22.56±3.41	None	200μg/kg over 24 h	Placebo	In-hospital
Sharma 2014^30^	40	CABG+mitral valve repair	23.55±4.87	22.55±0.92	None	200μg/kg over 24 h	Placebo	In-hospital
Tritapepe 2006^31^	24	CABG	50±7	52±5	24μg/kg	None	Placebo	In-hospital
Tritapepe 2009^32^	102	CABG	41.6±10.7	44.1±9.8	24μg/kg	None	Placebo	In-hospital

LVEF: left ventricular ejection fraction; CABG: coronary artery bypass grafting; HF: heart failure; ADHF: acutely decompensated heart failure; OPCABG: Off-pump coronary artery bypass surgery; STEMI: ST-segment elevation infarction; AVR: aortic valve replacement; LCOS: low cardiac output syndrome All studies considered values < 0.05 to indicate statistical significance.

### Study quality and publication bias

Twenty studies were randomized using methods including computer-generated randomization schedules or random number tables, and the randomization process was documented. In 22 studies, allocation concealment was documented with low bias risk, yet this was questionable in seven others. In 73% of the studies, double-blinding was utilized, providing effective blinding to limit participants’ or investigators’ bias on adverse reaction reports. The bias risk assessment results are presented in Figure 2. Figure 3 illustrates the publication bias risk in the included studies. Egger’s test revealed no publication bias evidence (p=0.251).

**Figure 2:** – Risk of bias assessment of the included studies.

**Figure 3:** – The risk of publication bias for the included studies.

### Levosimendan and atrial fibrillation incidence

Eleven studies assessed the non-postoperative AF occurrence and stated that levosimendan elevated the non-postoperative AF risk significantly (Central Illustration). Eighteen studies reported that levosimendan reduced postoperative AF incidence significantly (Central Illustration). Moreover, the AF incidence decreased significantly more in patients who used levosimendan after cardiac surgery than those who used levosimendan before cardiac surgery (Table 2). Heterogeneity was mostly due to differences in inclusion criteria, levosimendan dose, LVEF, cardiac surgery, and follow-up duration.

**Table 2:** – Subgroup analyses

Group	No. studies	Heterogeneity		Model	Meta-analysis	
		p	I^**2**^ (%)		OR(95%CI)	p
**Levosimendan vs Placebo/Control**						
LVEF≤40%	20	<0,001	71,6	Aleatório	0,82 (0,55, 1,22)	0,33
LVEF>40%	4	0.545	0	Aleatório	1,30 (0,80, 2,13)	0,30
**Time of administration**						
Before surgery	14	<0,001	75,3	Aleatório	0,67 (0,42, 1,06)	0,09
After surgery	4	0,870	0	Aleatório	0,53 (0,32, 0,88)	0,02
Follow-up duration						
≤7 days	18	<0,001	68,5	Aleatório	0,68 (0,42,1.12)	0,14
>7 days	11	0.812	0	Aleatório	1,30 (1,07, 1,57)	0,007
**Bolus dose**						
≥12 μg/kg	10	0.294	16.2	Aleatório	1,44 (1,10, 1,88)	0,004
<12 μg/kg	9	<0,001	72,6	Aleatório	0,64 (0,34, 1,20)	0,16


LVEF: left ventricular ejection fraction.

### Subgroup analysis

A subgroup analysis was conducted according to the LVEF: LVEF≤40% and LVEF>40%. Levosimendan did not significantly change the incidence of AF in both subgroups (Table 2).

Levosimendan was given as a bolus dose followed by continuous infusion in 14 studies, while only continuous infusion was utilized in 13 studies in this meta-analysis. The bolus dose ranged from 6 to 24 μg/kg, and the continuous infusion dose ranged from 0.1 to 0.4 μg/kg/min. The levosimendan bolus dose effect on the AF risk was evaluated by conducting another subgroup analysis. The participants were classified into two subgroups: a large bolus dose (levosimendan dose≥12 μg/kg) and a small bolus dose (levosimendan dose<12 μg/kg) with 0.1 and 0.2 μg/kg/min as a continuous infusion. The AF incidence was significantly increased by a large bolus dose and decreased by a small bolus dose (Table 2).

Analyzing the follow-up length, levosimendan was significantly linked to a high AF risk in eleven studies with more than seven days of follow-up. In 18 studies with no more than seven days of follow-up, a lower AF incidence in the levosimendan group in comparison with controls (Table 2).

### Sensitivity analysis

The pooled results were calculated. None of the studies influenced the pooled results and changed the conclusion of this analysis. The study pooled results were analogous (Figure 4).

**Figure 4:** – Sensitivity analysis.

## Discussion

As far as we know, this meta-analysis contained the largest number of RCTs, which compared levosimendan and placebo or other therapies and provided data on the AF occurrence during follow-up. As per our findings, high non-postoperative AF and low postoperative AF incidences were linked to levosimendan. We further found that patients who used levosimendan after cardiac surgery had a more pronounced decrease in the incidence of AF than those who used levosimendan before cardiac surgery.

Levosimendan is a calcium-sensitizer that augments the myocardial contractility by enhancing the myofilament sensitivity to calcium via binding to cardiac troponin C in a calcium-dependent manner.^34^ It also opens mitochondrial ATP-sensitive potassium channels in cardiac and vascular tissues and exerts peripheral vasodilatory and anti-ischemic effects.^35^ Common inotropic agents improve myocardial contractility by increasing Ca^2^ that can bind to cardiac troponin-C by increasing myocardial energy, oxygen demand, and the incidence of arrhythmia. Therefore, it has the potential to cause arrhythmias. Conversely, Levosimendan does not increase myocardial energy demand and oxygen consumption.^36^ It does not affect intracellular free calcium, therefore, the potential for arrhythmias is also reduced.

A previous meta-analysis did not find that levosimendan infusion could reduce the AF risk in patients undergoing cardiac surgery.^10^ The large-scale randomized clinical trials (LEVO-CTS, CHEETAH, and LICORN) also did not increase the AF incidence rate in the levosimendan group. Our findings demonstrated that levosimendan significantly decreased the AF risk following cardiac surgery and increased the incidence of non-postoperative AF. Levosimendan decreased aAPD_90_ and aERP significantly. AF reentry and stability are affected by reducing the action potential duration and refractory period.^37^ The cardiac surgery characteristics differ from levosimendan in acute HF patients. The factors applicable to heart disease patients are usually different from those applicable to the perioperative period. The suspension of standard perioperative treatment, blood loss, cardiopulmonary bypass (CPB) usage, intravascular and extravascular fluids rapid transfer, and systemic inflammatory response syndrome can influence the levosimendan therapy outcome. The metabolite OR-1896 of levosimendan forms slowly in the cardiac surgery environment compared with patients with HF. Peak levels were reached five to six days following the levosimendan infusion cessation because of the fasting following surgery and the broad-spectrum antibiotics usage in cardiac surgery.^38^ From the pharmacokinetic characteristics of levosimendan, its usage before or during the perioperative period may have very different effects. The levosimendan inotropic and cardioprotective characteristics can have a long-lasting effect, helping reduce postoperative complications. Experts advised using levosimendan for generally impaired myocardial function patients one day before cardiac surgery.^39^ However, the subgroup analysis showed that levosimendan usage after cardiac surgery is linked to a considerable decrease in AF incidence. The mechanism of levosimendan inhibiting postoperative AF is unclear, but it may be related to the levosimendan antioxidant and anti-inflammatory characteristics.^40^ However, there are currently no significantly high-quality trials using levosimendan at this time point, requiring further evaluation.

The AF incidence rate elevates with the HF severity.^41^ Harrison et al. conducted a meta-analysis comprising 14 randomized controlled trials, including 1,155 cardiac surgery patients, to explore the levosimendan safety and efficacy profile grouped by LVEF.^9^ The patients were classified into low or preserved-LVEF groups with a mean of LVEF< 40% or LVEF> 40%, respectively. Data analysis demonstrated a significant decrease in the levosimendan death risk, and the subgroup analysis showed that this was only confined to the low-LVEF studies. No benefit was observed in the “preserved-LVEF group”. Further analysis revealed that the “low-LVEF group” patients receiving levosimendan significantly decreased the postoperative AF incidence. The preserved-LVEF group showed no difference as well. Our findings demonstrated that levosimendan did not significantly change the AF incidence in LVEF>40% and LVEF≤40% subgroups.

A previous study showed that a 6-24 μg/kg bolus dose delivered in 10 min followed by a 0.05 to 0.2 μg/kg/min infusion rate is the optimum levosimendan dosing regimen.^42^ Papp Z et al. suggested another levosimendan regimen, a 6-12 μg/kg delivered in 10 min followed by 0.05 or 0.1 or 0.2 μg/kg/min for 24 h.^43^ Since dobutamine has a few minutes half-life, while levosimendan’s half-life is about one hour, the dobutamine hemodynamic effects can be observed immediately following the infusion, while the immediate effect can be observed only with a large dose of levosimendan. High doses of levosimendan were associated with arrhythmias in cases of hypovolemia or initial hypotension.^44,45^ Our findings demonstrated that a large levosimendan bolus dose (≥12 μg/kg) was linked to a high AF incidence, and a small levosimendan bolus dose (<12 μg/kg) was linked to a low AF incidence. Experts in the European consensus on levosimendan usage during the perioperative period do not recommend high-dose administration. When high-dose administration is required, most of them recommend reducing the dose.^39^

Nieminen et al. demonstrated that IV levosimendan administration for 24 h leads to dose-dependent hemodynamic effects stating a clear correlation between them. Its active protein-bound metabolites OR-1855 and OR-1896 exert clinical effects for up to seven days.^46^ The presence of the long-acting metabolite means that hemodynamic effects continue for a week following the levosimendan infusion, and the possibility of adverse reactions also increases with time. This research meta-analysis revealed that levosimendan was significantly linked to a high AF risk with more than seven days of follow-up. Levosimendan tended to increase the risk of AF when follow-up was less than or equal to seven days. A large randomized controlled study is warranted.

As far as we know, this is the first research to analyze the levosimendan and AF risk correlation based on the timing of administration, levosimendan dose, and the length of follow-up. We rigorously screened the literature and included all eligible RCTs containing levosimendan and AF data. This research had various limitations. First, some outcome assessments had moderate or high heterogeneity, so we did many subgroup analyses for every result to investigate the heterogeneity source. Additionally, we did a sensitivity analysis through high-bias risk studies exclusion, which did not change the main results. Second, because the sample size of some studies is very small, which does not bring enough evidence, large clinical trials with convincing evidence are necessary to solve this situation.

## Conclusions

High non-postoperative AF and low postoperative AF were linked to levosimendan, according to the meta-analysis conducted in this study. Our findings demonstrated that a large bolus dose of levosimendan was linked to a high AF incidence, and a small bolus levosimendan dose was linked to a low AF incidence. The levosimendan usage was considerably linked to a high AF risk with more than seven days of follow-up. Levosimendan tended to increase the risk of AF when follow-up was less than or equal to seven days. Further high-quality clinical studies are necessary to confirm our findings.
